# Paclitaxel induces nucleolar enlargement in dorsal root ganglion neurons *in vivo* reducing oxaliplatin toxicity

**DOI:** 10.1038/sj.bjc.6601012

**Published:** 2003-06-10

**Authors:** S M F Jamieson, J Liu, T Hsu, B C Baguley, M J McKeage

**Affiliations:** 1 Department of Pharmacology and Clinical Pharmacology, The University of Auckland, Private Bag 92019, Auckland, New Zealand; 2 Auckland Cancer Society Research Centre, The University of Auckland, Private Bag 92019, Auckland, New Zealand

**Keywords:** paclitaxel, oxaliplatin, neurotoxicity, pharmacokinetics, dorsal root ganglion, cell nucleolus, combination chemotherapy

## Abstract

Paclitaxel and oxaliplatin are promising drugs for combination trials but both induce peripheral neurotoxicity. To investigate this toxicity, 10-week-old female Wistar rats were given single intraperitoneal doses of paclitaxel and oxaliplatin, alone or in combination. Neurotoxicity was assessed by L5 dorsal root ganglion morphometry and H-reflex-related sensory nerve conduction velocity. Platinum concentrations in dorsal root ganglia and plasma were measured by inductively coupled plasma mass spectrometry. Dorsal root ganglion nucleolus size was significantly increased following single doses of paclitaxel of 10 and 20 mg kg^−1^ at 24 h and 6 days (*P*<0.02). In contrast, dorsal root ganglion nucleolus size was significantly decreased following single doses of oxaliplatin ranging from 3 to 30 mg kg^−1^ at time points ranging from 2 h to 14 days. Sensory nerve conduction velocity was altered after a single dose of oxaliplatin but not after paclitaxel. In combination with oxaliplatin, paclitaxel did not alter the plasma pharmacokinetics or dorsal root ganglion accumulation of oxaliplatin-derived platinum. However, prior paclitaxel inhibited oxaliplatin-induced reductions of dorsal root ganglion nucleolar diameter (*P*<0.02). Sensory nerve conduction velocity was reduced after oxaliplatin alone (*P*<0.05) but unchanged when paclitaxel was given before oxaliplatin. In conclusion, paclitaxel induces nucleolar enlargement in dorsal root ganglion neurons after pharmacologically relevant doses *in vivo* and reduces oxaliplatin nucleolar damage and neurotoxicity.

Peripheral sensory neurons are a heterogeneous group of postmitotic cells (reviewed in Perl, 1992) that are damaged by chemotherapy drugs. The cell bodies of peripheral sensory neurons are contained within the dorsal root ganglia, and their axons extend for large distances to innervate peripheral tissues and the central nervous system. The cell bodies and peripherally directed axons of dorsal root ganglion neurons lack the protection of the blood–brain barrier, and are exposed to neurotoxins to a greater extent than the central nervous system. For example, the dorsal root ganglion is the main site of platinum accumulation during treatment with platinum-based antitumour drugs (Gregg *et al*, 1992; Screnci *et al*, 2000).

Dorsal root ganglion neurons have a large nucleolus, evident as a prominent dark-staining subnuclear structure by light microscopy. The nucleolus is the cellular site of rDNA gene transcription and processing of preribosomal RNA transcripts (Shaw and Jordan, 1995). The prominence of the nucleolus of dorsal root ganglion neurons presumably reflects a high requirement for ribosomal gene transcription, ribosome production and protein synthesis to maintain their large cell body and extensive axonal projections. The neurotoxicity of platinum-based drugs has been previously linked to damage to the nucleolus of dorsal root ganglion neurons (Tomiwa *et al*, 1986; Muller *et al*, 1990; Cavaletti *et al*, 1992,^1998^; Cece *et al*, 1995; Stacchiotti *et al*, 1995; Holmes *et al*, 1998). For example, we have previously shown that the neurotoxicity of a series of platinum-based drugs correlates with the rate of shrinkage of dorsal root ganglia nucleoli during multiple-dose treatment (McKeage *et al*, 2001). Less is known about the dorsal root ganglia nucleolar effects of paclitaxel, an anticancer drug whose clinical application is also limited by peripheral neuropathy.

Paclitaxel and oxaliplatin are showing promising activity given in combination clinical trials in drug refractory ovarian cancer (Faivre *et al*, 1999; Delaloge *et al*, 2000). Their mechanisms of action involve the arrest of cell progression through the mitotic cycle and cytotoxicity to proliferating tumour cells, respectively (Raymond *et al*, 1998; Blagosklonny and Fojo, 1999). The clinical application of this drug combination has been limited by neurotoxicity possibly due to damage to dorsal root ganglion neurons. This toxicity is not readily explained by their mechanisms of action since dorsal root ganglion neurons are postmitotic cells and not susceptible to antiproliferative toxicity. With these considerations, it seemed reasonable to investigate the effects of paclitaxel on dorsal root ganglia *in vivo,* given alone and in combination with oxaliplatin.

## METHODS

### Animals and treatment

Female Wistar rats (10-week-old, age-matched) were used that weighed between 200 and 300 g at the start of the experiments, and were acclimatised to handling prior to drug treatment. Paclitaxel (Phytogen Life Sciences Inc., Delta, BC, Canada) was prepared by solubilisation in Cremophor EL (Sigma, St Louis, MO, USA)/ethanol/0.9% NaCl (Baxter Healthcare, Old Toongabbie, Australia) by vortexing and sonication at an injection volume of 10 ml kg^−1^ body weight. Oxaliplatin (Sanofi Winthrop, France) was dissolved in 0.9% NaCl, vortexed and sonicated, and administered to the animals at an injection volume of 10 ml kg^−1^. In each case, control animals were administered the relevant drug vehicle alone. All drug treatments were administered by intraperitoneal injection. When the drugs were given in combination, paclitaxel was given 24 h before oxaliplatin, and dorsal root ganglia parameters and sensory nerve conduction velocity were measured at 24 h and 3 weeks after dosing of oxaliplatin, respectively. All animal procedures and use complied with ethical guidelines and were approved by the Animal Ethics Committee of the University of Auckland and met the requirements of the UKCCCR guidelines. Animals were maintained in a constant environment and had unrestricted access to food and water. Animals were checked daily for signs of toxicity. Any animals suffering visible toxicity were immediately euthanised.

### Dorsal root ganglion morphometry

Animals were anaesthetised with 0.9 ml of 3 mg ml^−1^ pentobarbitone (Chemstock Animal Health Ltd., Christchurch, New Zealand). Following the induction of deep anaesthesia, intracardiac paraformaldehyde perfusion was carried out by administering 60 ml of 0.9% NaCl, followed by 60 ml of 4% paraformaldehyde in 0.1 M phosphate buffer. L5 dorsal root ganglia were carefully dissected out and stored in 4% paraformaldehyde. Dorsal root ganglia were washed in water, dehydrated in a series of alcohols, cleared in xylene and embedded in paraffin wax. A microtome was then used to cut each dorsal root ganglion into 6 *μ*m sections. Typically, about 60–80 sections were produced for each dorsal root ganglion. Sections were mounted on slides and stained with haematoxylin and eosin. A total of 10 sections selected at random regular intervals were analysed from each dorsal root ganglion by light microscopy at × 630 magnification. Images were digitally photographed to a computer by an Axiocam camera (Carl Zeiss Vision). Area or perpendicular diameters were measured for the cell body, nucleus and nucleolus of the two to five largest cells of each section by AxioVision 3.0 (Carl Zeiss Software) software. Morphometric parameters from the 10 largest dorsal root ganglion nerve cells were averaged to provide the cell body, nucleus and nucleolus measurements for each dorsal root ganglion.

### Sensory nerve conduction velocity (SNCV) recordings

Recordings of evoked H-plantar responses following nerve stimulation at the ankle and sciatic notch were used to calculate SNCV as described previously (McKeage *et al*, 1994). Briefly, animals were anaesthetised with intramuscular Hypnorm (Janssen Pharmaceutica, Beerse, Belgium) diluted 1 : 1 in milli-Q water. Percutaneous needle electrodes were used to generate responses by electrically stimulating the sciatic nerve at the sciatic notch and the tibial nerve at the ankle of the left hind limb. H- and M-waves were recorded through differential silver/silver chloride electrodes fixed to the sole and dorsum of the left hind leg. SNCV was calculated by dividing the distance between the sites of stimulation by the difference in evoked H-response latencies between the ankle and sciatic notch after stimulation.

### Plasma and tissue platinum determinations

At 0, 15, 30, 45 min, 1, 2, 4 and 24 h after the administration of oxaliplatin, blood samples (approximately 100 *μ*l) were taken from the tail artery of each animal into tubes containing heparin diluted with 0.9% NaCl. Methanol extracts of plasma were prepared by centrifugation of whole blood at 5000 r.p.m. for 10 min, followed by addition of an equal volume of ice-cold methanol to plasma and two further centrifugation steps at 14 000 r.p.m. for 15 min. Plasma samples were stored at −20°C prior to analysis by inductively coupled plasma mass spectrometry. On the day of analysis, methanol extracts of plasma were diluted with 1% nitric acid to give a volume of 2 ml per sample. Samples were then analysed using an HP 4500 ICP-MS (Hewlett-Packard, Yokowaga, Japan). The operating conditions were the same as those outlined previously (Screnci *et al*, 1998). At 24 h after drug administration, animals were anaesthetised using 0.9 ml of 3 mg ml^−1^ pentobarbitone and exsanguinated. L4, L5 and L6 dorsal root ganglia were dissected out and prepared for ICP-MS analysis as described previously (Screnci *et al*, 1998). Briefly, tissues were left overnight in a 1 ml solution of 70% nitric acid, before digestion for 2 h at 90°C in a sand bath. On the day of analysis, each sample was made up to 10 ml by addition of milli-Q water and then introduced into the ICP-MS. Plasma and tissue platinum content was determined from standards made up in the relevant matrix.

### Statistical analysis

The statistical significance of differences between means and trends was evaluated by 95% confidence intervals (95% CI), unpaired *t*-tests, one- or two-way analysis of variance (ANOVA) and regression analysis using Prism 3.0. software (GraphPad Software, CA, USA). In each case, a *P*-value of <0.05 or nonoverlapping 95% CI was regarded as indicating statistical significance.

## RESULTS

### Age-dependent changes in experimental parameters

Significant age-dependent increases in body weight (0.45% day^−1^; *P*<0.0001; two-way ANOVA), sensory nerve conduction velocity (0.19% day^−1^; *P*<0.0001; two-way ANOVA), dorsal root ganglion cell body area (0.8% day^−1^; *P*<0.01; two-way ANOVA) and dorsal root ganglion nucleolar area (0.3% day^−1^; *P*<0.01; two-way ANOVA) occurred during the experiment independent of treatment group allocation. There was no significant time-dependent change in dorsal root ganglion nuclear area.

### Paclitaxel and oxaliplatin alone

**Figure 1:**
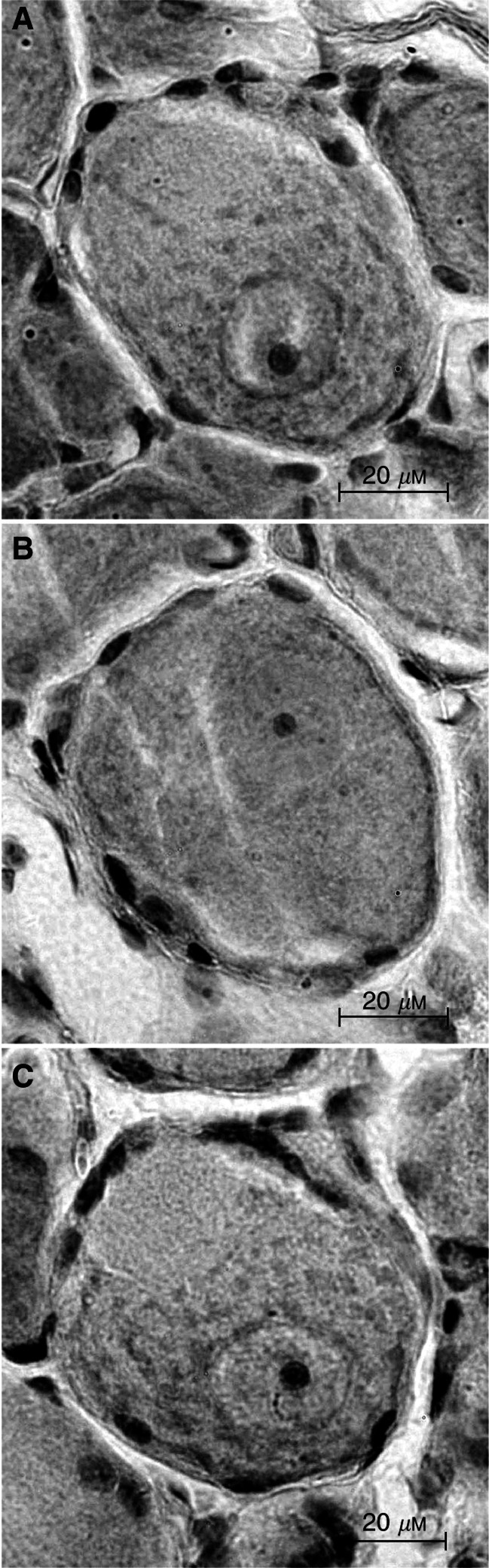
Photomicrographs of rat L5 dorsal root ganglia after treatment with paclitaxel (**A**), oxaliplatin (**B**) or control (**C**). Paclitaxel and oxaliplatin were given as single i.p. doses of 10 mg kg^−1^. Dorsal root ganglia were collected 24 h after treatment. Nucleolus size appeared increased and decreased after treatment with paclitaxel and oxaliplatin, respectively. Magnification, × 630, bar represents 20 *μ*m.

The effect of paclitaxel on the morphometry of L5 dorsal root ganglia of rats was studied. A single dose of 10 mg kg^−1^ of paclitaxel was well tolerated, but 20 mg kg^−1^ caused deterioration in general condition approximately 1 week after dosing necessitating euthanasia. There was no significant treatment-related change on L5 dorsal root ganglia cell body or nucleus size after single-dose paclitaxel. However, nucleolar size was significantly increased following single doses of 10 and 20 mg kg^−1^, at 24 h (*P*<0.02) and 6 days (*P*<0.02) (Figures 1

and 2A

**Figure 2:**
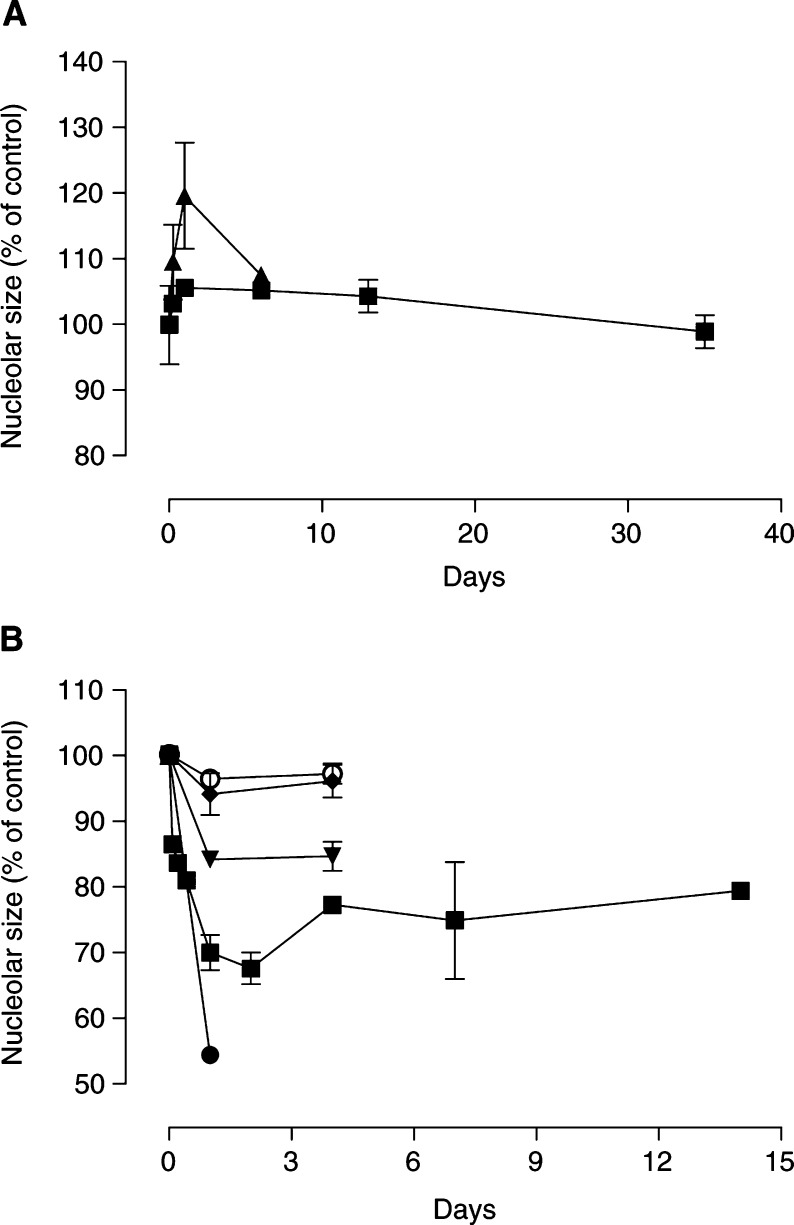
Dorsal root ganglion nucleolus size after treatment with paclitaxel (**A**) or oxaliplatin (**B**). Paclitaxel and oxaliplatin were given as a single i.p. dose of 0.3 mg kg^−1^ (○), 1 mg kg^−1^ (⧫), 3 mg kg^−1^ (▾), 10 mg kg^−1^ (▪), 20 mg kg^−1^ (▴) or 30 mg kg^−1^ (•) on day 0. Nucleolus diameter was expressed as the percentage of control. Control values were determined at each time point. Symbols represent average values (*n*=2–4) and the s.d.

). Nucleolus size returned to control values after 14 days. Nucleolus size showed a linear dependence upon paclitaxel dose at 6 h (*r*^2^=0.54, *P*=0.004), 24 h (*r*^2^=0.79, *P*=0.0001) and 6 days (*r*^2^=0.70, *P*=0.0001) after dosing.

The morphometry of L5 dorsal root ganglia was also studied after treatment with oxaliplatin. Single doses of oxaliplatin up to 10 mg kg^−1^ were well tolerated, but 30 mg kg^−1^ caused deterioration in general condition within a few days of dosing necessitating euthanasia. There was no significant change in cell body or nucleus size but nucleolus size was decreased within a few hours of giving oxaliplatin (Figures 1 and 2B). Reductions of nucleolus size were maximal at 24–48 h and persisted for at least 2 weeks after dosing. Nucleolus size showed a nonlinear dependence on oxaliplatin dose at 24 h after treatment (*r*^2^=0.99). The dose curve showed a maximum reduction of nucleolus size at 53.7% of control (95% CI, 51.9–55.5). The oxaliplatin dose required for half-maximal reduction in nucleolus size was 4.24 mg kg^−1^ (95% CI, 3.67–5.02).

**Figure 3:**
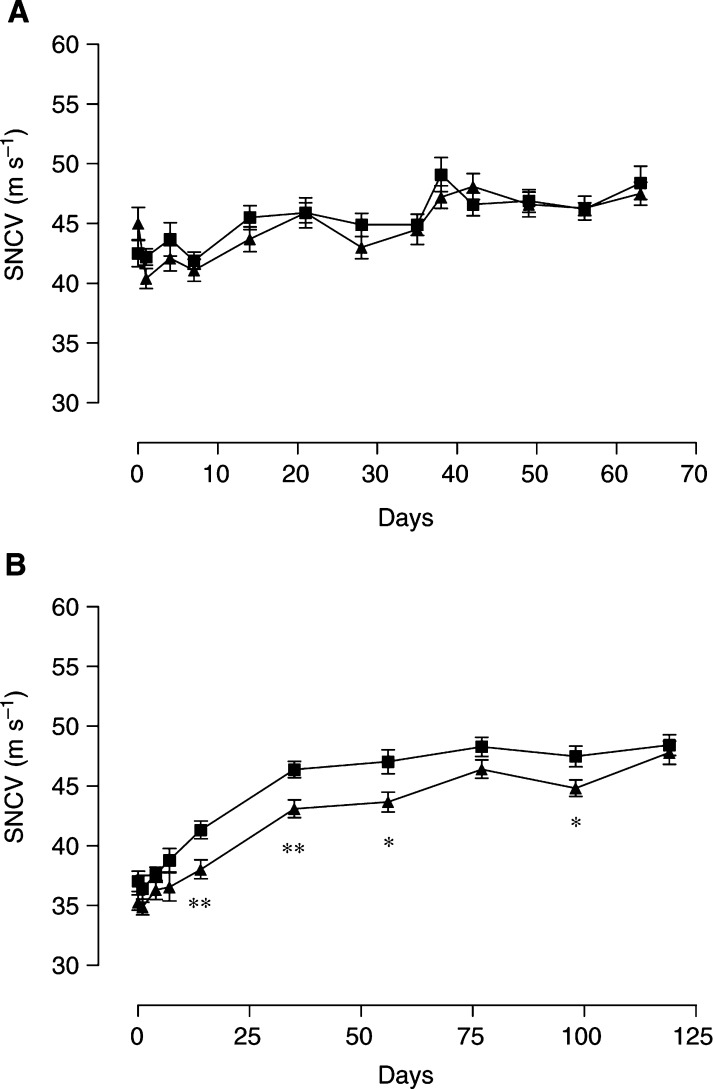
Nerve conduction velocity after treatment with paclitaxel (**A**) or oxaliplatin (**B**). Paclitaxel and oxaliplatin were given as single i.p. doses of 10 mg kg^−1^ (▴) on day 0. Controls were given the corresponding drug vehicle (▪) on day 0. Sensory nerve conduction velocity was measured at various times after treatment. Symbols represent averaged values (*n*=20–25) and the s.e.m. ^**^, *P*<0.005; ^*^, *P*<0.05.

To determine the functional significance of these nucleolar changes, nerve conduction velocity was measured in rats at various times after treatment with single doses of paclitaxel or oxaliplatin. A single dose of paclitaxel of 10 mg kg^−1^ had no effect on sensory nerve conduction velocity (Figure 3A

). However, sensory nerve conduction velocity was significantly reduced after a single 10 mg kg^−1^ dose of oxaliplatin (Figure 3B). Nerve conduction velocity became altered at 14 days after oxaliplatin treatment and remained reduced until returning to control values at 120 days postdosing. Thus, sensory nerve conduction velocity became altered following the onset of oxaliplatin-induced nucleolar shrinkage, but was unchanged in association with or following paclitaxel-induced nucleolar enlargement.

### Paclitaxel and oxaliplatin in combination

**Figure 4:**
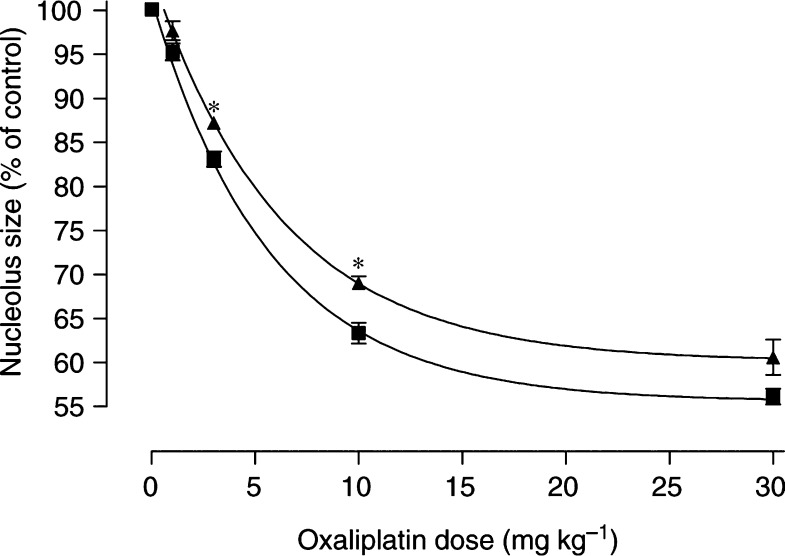
Dorsal root ganglia nucleolus size after oxaliplatin alone (▪) or oxaliplatin given in combination with paclitaxel (▴). Paclitaxel was given as a single i.p. dose of 20 mg kg^−1^. Oxaliplatin was given 24 h later as single i.p. doses of 1–30 mg kg^−1^. Dorsal root ganglia were collected 24 h after oxaliplatin dosing to determine nucleolus size. Symbols represent averaged values (*n*=2–6) and the s.e.m. ^*^, *P*<0.02.

Since oxaliplatin and paclitaxel had opposite effects on dorsal root ganglion nucleolus size, combination treatment was next studied. To investigate the early-onset nucleolus changes, a single dose of paclitaxel (20 mg kg^−1^) was given 24 h before single doses of oxaliplatin ranging from 1 to 30 mg kg^−1^. Dorsal root ganglia were collected 24 h after oxaliplatin treatment to determine nucleolus size. Nucleolus size was significantly increased (*P*<0.02) after combination treatment with paclitaxel and oxaliplatin, compared with oxaliplatin alone, at the 3.3 and 10 mg kg^−1^ oxaliplatin dose levels (Figure 4

). Nucleolus size was also numerically increased after combination treatment at the 30 mg kg^−1^ oxaliplatin dose level, although this difference did not reach statistical significance (*P*=0.058). As before, nucleolus size decreased with a nonlinear dependence on oxaliplatin dose (*r*^2^=0.99). Based on the parameters of the fitted curve, the oxaliplatin dose required for half-maximal reduction in nucleolus size (3.98 mg kg^−1^ (95% CI, 3.38–4.66)) was not altered by giving paclitaxel (4.32 mg kg^−1^ (95% CI, 3.53–5.58)). However, the maximal reduction in nucleolus size was significantly altered by giving paclitaxel with oxaliplatin (60.1% of control (95% CI, 57.7–62.5)) compared with giving oxaliplatin alone (55.6% of control (95% CI, 54.0–57.1)).

**Figure 5:**
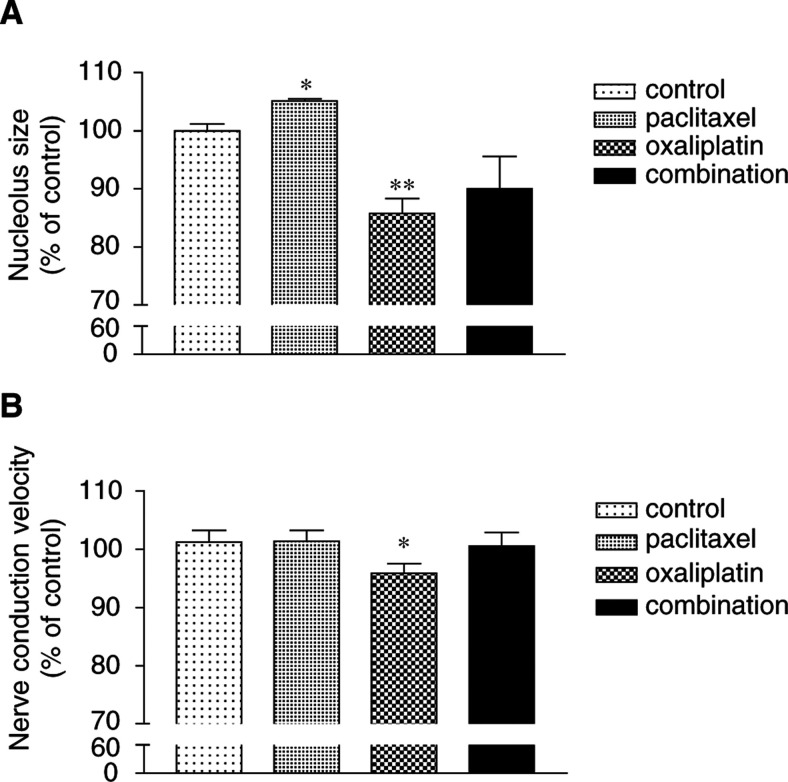
Dorsal root ganglion nucleolus size (**A**) and nerve conduction velocity (**B**) after paclitaxel and oxaliplatin given alone or in combination. Paclitaxel was given as a single i.p. dose of 10 mg kg^−1^. After 24 h later, oxaliplatin was given as a single i.p. dose of 10 mg kg^−1^. Drug vehicle was given to controls at corresponding times. Dorsal root ganglia were collected for determining nucleolus size at 24 h after oxaliplatin dosing. Sensory nerve conduction velocity was measured 3 weeks after oxaliplatin dosing. Bars represent the mean and the s.e.m. *n*=3–4 for nucleolus size, *n*=23–25 for nerve conduction velocity; ^*^, *P*<0.05; ^**^, *P*<0.01.

The effect of single doses of paclitaxel of 10 mg kg^−1^ in combination with oxaliplatin was next studied. Paclitaxel (10 mg kg^−1^) was given 24 h prior to oxaliplatin (10 mg kg^−1^). Nucleolus size was measured 24 h after oxaliplatin dosing. Sensory nerve conduction velocity was measured 3 weeks after treatment. To account for interoccasion variation in experimental parameters between different batches of animals, data were expressed as percentage of control. Nucleolus size was increased after paclitaxel alone (*P*<0.05), decreased after oxaliplatin alone (*P*<0.01) but not significantly altered after giving paclitaxel and oxaliplatin in combination (Figure 5A

). There was a significant reduction in sensory nerve conduction velocity in the oxaliplatin-alone group (*P*<0.05) indicating the presence of functional neurotoxicity (Figure 5B). However, sensory nerve conduction velocity in the other treatment groups, given paclitaxel alone or paclitaxel combined with oxaliplatin, was similar to the control group.

The possibility of a pharmacokinetic interaction being a basis for the reduction in oxaliplatin toxicity was next investigated. Paclitaxel (10 mg kg^−1^), or its drug vehicle, were given 24 h before oxaliplatin (10 mg kg^−1^). Blood was then collected at various times over 24 h and lumbar dorsal root ganglia were collected 24 h after dosing. The platinum content of dorsal root ganglia and deproteinised plasma was determined by ICP-MS. The AUC of unbound plasma oxaliplatin-derived platinum (33.0±2.39 ng h l^−1^) was not significantly altered by giving paclitaxel (42.4±4.55 ng h l^−1^; *P*=0.1). Similarly, the platinum content of lumbar dorsal root ganglia 24 h after oxaliplatin treatment (1.30±0.10 ng mg^−1^) was not significantly altered by giving paclitaxel (1.47±0.13 ng mg^−1^; *P*=0.33). Thus, under conditions associated with reduction of oxaliplatin toxicity, there was no significant reduction in the concentrations of oxaliplatin-derived platinum in the plasma or dorsal root ganglia.

## DISCUSSION

In the present study, the effects of the systemic administration of paclitaxel were investigated at pharmacologically relevant doses *in vivo.* Morphometric analysis of L5 dorsal root ganglia revealed enlargement of the nucleoli in the cell bodies of sensory neurons after a single-dose treatment. Time-dependent changes in dorsal root ganglion morphometric parameters and sensory nerve conduction velocity were also documented that occurred independent of paclitaxel treatment and might have resulted from the growth of animals during the experiments. Despite the changing baselines, significant increases in nucleolus size were found in association with paclitaxel treatment at 24 h and 6 days after a single dose. At 24 h after doses of 10 and 20 mg kg^−1^, averaged nucleolar diameters increased by 5.6 and 19.6% relative to control, respectively. Thus, the systemic administration of paclitaxel was associated with nucleolar enlargement in dorsal root ganglion neurons after pharmacologically relevant doses *in vivo*.

The cell bodies of peripheral nerves are known to undergo nucleolar enlargement, in conjunction with other responses, after damage to their peripherally directed axons (reviewed in Lieberman, 1971). For example, dorsal root ganglion neurons develop an increase in nucleolar size after crush injuries to the sciatic nerve (Wells and Vaidya, 1989). Like other antimicrotubule agents, paclitaxel is known to damage the axons of peripheral nerves (Cavaletti *et al*, 1995,^1997^; Authier *et al*, 2000; Mimura *et al*, 2000). Direct injection of paclitaxel into peripheral nerves has also been shown in one previous study to cause nucleolar enlargement in the corresponding nerve cell bodies (Nennesmo and Reinholt, 1988). These considerations might suggest that the nucleolar enlargement demonstrated in the present study is due to a retrograde response of dorsal root ganglion cell bodies to peripheral axon injury. However, the effect of paclitaxel on dorsal root ganglion nucleoli in this study occurred independent of paclitaxel-induced axonal damage that was detectable by changes in sensory nerve conduction velocity.

Other mechanisms could therefore be involved in the nucleolar enlargement caused by paclitaxel. Fluorescent paclitaxel derivatives have been shown to bind avidly to the nucleoli of interphase cells (Guy *et al*, 1996; Rao *et al*, 1998) and binding of nucleolar proteins by exogenous substances can alter the size and composition of the nucleolus (Abadia-Molina *et al*, 1998). Paclitaxel has also been shown to induce the expression of numerous genes (Moos and Fitzpatrick, 1998; Fukusaki *et al*, 2001) at concentrations possibly achieved under the conditions used in the present study. Changes in gene expression may be the basis for paclitaxel effects on nucleolus size since this is the site of transcription of rDNA genes (Shaw and Jordan, 1995) and variations in nucleolar dimensions are closely linked to changes in rRNA synthesis (Goessens, 1984).

Oxaliplatin also altered dorsal root ganglion nucleolus size. Significant reductions of nucleolus diameter occurred after single doses of oxaliplatin at a range of doses and time points. The changes in nucleolus size induced by oxaliplatin were in the opposite direction compared to those induced by paclitaxel. Alterations in dorsal root ganglion nucleoli appear to be linked with the neurotoxicity of platinum-based drugs. For example, we have previously shown that the neurotoxicity of platinum-based drugs correlates with the rate of dorsal root ganglion nucleolus shrinkage during multiple-dose drug treatment (McKeage *et al*, 2001). Several groups have reported that shrinkage of dorsal root ganglion nucleoli is associated with cisplatin (Tomiwa *et al*, 1986; Muller *et al*, 1990; Cavaletti *et al*, 1992; Cece *et al*, 1995; Stacchiotti *et al*, 1995), carboplatin (Cavaletti *et al*, 1998) and ormaplatin (Holmes *et al*, 1998). The change in nucleolus size may come about by the inhibition of rRNA synthesis, which is a known effect of platinum-based drugs (Jordan and Carmo-Fonseca, 1998).

Since oxaliplatin and paclitaxel had opposing effects on dorsal root ganglion nucleolar size, they were also studied in combination. Prior paclitaxel significantly inhibited the reductions in nucleolus size and nerve conduction velocity induced by a single dose of oxaliplatin. The inhibitory effect of paclitaxel was not explained by a pharmacokinetic interaction. Paclitaxel did not significantly alter the plasma AUC for unbound oxaliplatin-derived platinum or the levels of oxaliplatin-derived platinum in dorsal root ganglia 24 h after treatment. In fact, there were nonsignificant trends towards higher platinum levels in the combination group that would have been expected to increase rather than reduce oxaliplatin toxicity. A pharmacodynamic interaction is therefore likely to have been involved in the paclitaxel inhibition of oxaliplatin neurotoxicity. Dorsal root ganglion neurons are well known for regenerating after peripheral nerve injuries (Bradbury *et al*, 2000). After peripheral nerve damage, dorsal root ganglia neurons show augmented recovery from a subsequent and more central injury (Oblinger and Lasek, 1984; Richardson and Issa, 1984; Chong *et al*, 1999). In this way, paclitaxel-induced axon damage may condition dorsal root ganglion neurons into a state of lower susceptibility to oxaliplatin toxicity without altering its pharmacokinetics.

In contrast to the present study, clinical trials of paclitaxel and oxaliplatin in combination have shown frequent and limiting neurotoxicity (Faivre *et al*, 1999; Delaloge *et al*, 2000). However, the conditions used in these clinical trials were not optimal for reducing neurotoxicity by this mechanism. For instance, we have previously shown minimisation of neurotoxicity when taxane drugs are given before platinum-based drugs but not with the reverse sequence of drug administration (McKeage *et al*, 1999). In two of the clinical trials, oxaliplatin was given before paclitaxel (Faivre *et al*, 1999; Liu *et al*, 2002), a dosing sequence not expected to show reduced neurotoxicity based on our previous work. Inhibition of neurotoxicity may also depend upon the kinetics of the onset of the paclitaxel effect on dorsal root ganglion neuronal nucleoli. In the present study, nucleolar enlargement was maximal 24 h after paclitaxel dosing, and giving paclitaxel 24 h before oxaliplatin inhibited oxaliplatin neurotoxicity. A delay between giving the two drugs may be required for the development of a cell body response to paclitaxel-induced axonal damage. In the clinical trials reported to date, paclitaxel and oxaliplatin were given sequentially without any dosing interval between giving the drugs (Faivre *et al*, 1999; Delaloge *et al*, 2000; Liu *et al*, 2002). Further clinical trials could be envisaged where paclitaxel was given 24 h before oxaliplatin, with neurotoxicity evaluation by symptom grading and electrophysiology carried out within a few days of the first treatment (Wilson *et al*, 2002). If evidence of reduced neurotoxicity was obtained, clinical trials could investigate whether minimisation of neurotoxicity is sustained during multiple-dose combination treatment with paclitaxel and oxaliplatin.

In conclusion, the systemic administration of paclitaxel, at pharmacologically relevant doses, caused nucleolar enlargement in dorsal root ganglion neurons *in vivo.* The mechanism of nucleolar enlargement may involve a response of the dorsal root ganglion cell body to a peripheral axonal injury. In contrast, oxaliplatin caused shrinkage of the dorsal root ganglion nucleoli *in vivo*. In combination with oxaliplatin, paclitaxel did not alter the plasma pharmacokinetics or dorsal root ganglion accumulation of oxaliplatin-derived platinum. However, paclitaxel inhibited oxaliplatin-induced dorsal root ganglia nucleolar damage and its neurotoxicity. The neurotoxicity of platinum–taxane combination chemotherapy may therefore depend upon opposing drug actions on the nucleolus of dorsal root ganglion neurons.
